# A time-duration measure of continuity of care to optimise utilisation of primary health care: a threshold effects approach among people with diabetes

**DOI:** 10.1186/s12913-019-4099-9

**Published:** 2019-05-02

**Authors:** Ninh Thi Ha, Mark Harris, David Preen, Suzanne Robinson, Rachael Moorin

**Affiliations:** 1 Health systems and Health economics, School of Public Health, Curtin University, Perth, Western Australia 6845 Australia; 2 School of Economics and Finance, Curtin University, Perth, Western Australia 6845 Australia; 3 Centre for Health Services Research, School of Population and Global Health, The University of Western Australia, 35 Stirling Highway, Crawley, WA 6009 Australia

**Keywords:** Cover index, Continuity of care, Optimal time interval, Diabetes mellitus, Primary care, Potentially preventable hospitalisation

## Abstract

**Background:**

Literature highlighted the importance of timely access and ongoing care provided at primary care settings in reducing hospitalisation and health care resource uses. However, the effect of timely access to primary care has not been fully captured in most of the current continuity of care indices. This study aimed to develop a time-duration measure of continuity of primary care (“cover index”) capturing the proportion of time an individual is under the potentially protective effect of primary health care contacts.

**Methods:**

An observational study was conducted on 36,667 individuals aged 45 years or older with diabetes mellitus extracted from Western Australian linked administrative data. Threshold effect models were used to determine the maximum time interval between general practitioner (GP) visits that afforded a protective effect against avoidable hospitalisation across complication cohorts. The optimal maximum time interval was used to compute a cover index for each individual. The cover was evaluated using descriptive statistics stratified by population socio-demographic characteristics.

**Results:**

The optimal maximum time between GP visits was 9–13 months for people with diabetes with no complication, 5–11 months for people with diabetes with 1–2 complications, and 4–9 months for people with diabetes with 3+ complications. The cover index was lowest among those aged 75+ years, males, Indigenous people, socio-economically disadvantaged and those in very remote areas.

**Conclusions:**

This study developed a new measure of continuity of primary care that adds a time parameter to capturing longitudinal continuity. Cover has the potential to better capture underuse of primary care and will significantly contribute to the sparsely available methods for analysis of linked administrative data in evaluating continuity of care for people with chronic conditions.

## Background

Given current pressures experienced by most health systems improvements in care delivery are needed to make the system more effective, efficient and sustainable. Over recent years the focus in many countries has been the enhancement of primary health care to reduce potentially preventable hospitalisations (PPH) which are often costly and undesirable for patients [52]. The rationale behind this is that timely utilisation and effective treatment in primary health care (PHC) settings for people with chronic conditions could afford a protective effect in preventing complications and adverse health events [10, 47]. For common chronic conditions such as diabetes, heart failure and asthma, a shift in focus from acute to primary care has the potential to delay or even prevent the onset of complications and reduce PPH. This theory surrounding ‘ambulatory care sensitive condition’ has been the driver of many policies aimed at increasing long-term ongoing, rather than sporadic or episodic, contact with a General Practitioner (GP).

The Australian government has set a focus on strengthening the PHC system to address inequities and future challenges of chronic diseases [15]. One of the ways this is being undertaken is by providing financial incentives for aspects of PHC and general practitioner (GP) behaviour, such as the introduction of Primary Health Networks, Integrated Care Models, Service/Practice Incentive Payments, Health Care Homes, Chronic Disease Management Medicare Benefits Scheme items and Home Medication Reviews [39]. Although GPs act as the gatekeeper of the health care system in Australia, it is not required that individuals register with a single practitioner or general practice. People are free to visit any GP they wish and can visit multiple GPs and general practices simultaneously. The role of GPs has been emphasised that GPs are the only physicians appropriate for taking the leading roles in the primary health care team and coordinating with other health care professionals into providing the best patient centred care including diagnosis, treatment and management [5].

Continuity of care (COC) is an important component of high-quality primary care as it is associated with increased patient satisfaction, quality of life and health outcomes [29, 42, 45, 49]. New models of care often rely on the theoretical link between COC and better health outcomes. A well-known conceptual framework of continuity of care proposed that continuity of care is a combination of three essential components: interpersonal continuity, management continuity and informational continuity [29, 32]. Interpersonal continuity is defined as an ongoing relationship between a patient and the same provider where the relationship between patients and providers are strengthen through mutual familiarity and personal trust [32]. Informational continuity is a link between providers to share comprehensive information about patients’ history of care and circumstances that helps to reduce duplicative and wasteful resources [32]. Management continuity is a collaboration between providers to ensure services delivered regularly and complementary and especially important in chronic and complex conditions which require management from multiple providers [32]. A sufficient continuity of care requires a presence of both care of an individual and proper management of care linked over time (Barker, Steventon, and Deeny 2017; [32]).

Although interpersonal continuity of care can be easily measured and widely used in literature [6, 7, 12, 18, 42], it is becoming more difficult to sustain due to the changing size of practices over time and to the recent evolution of large multi-partner (or corporate) practices rather than the solo-practice model common in previous decades (Gulliford, Naithani, and Morgan 2006; [32]). In the current context of a high burden of complex and multiple chronic conditions, health care for people with complex needs is now extended to a wide range of skills and settings to better manage chronic conditions [28, 29]. Thus, the view of continuity of care is concerned with management continuity - the extent of health care provided over time in a coordinated manner with appropriate response to patients’ needs [28]. While continuity of care is a complex multi-dimensional concept, current measures of continuity of care mostly reflect interpersonal continuity of care [6, 7, 36, 38]. Development of measures which can integrate management aspect of continuity would be useful to support comprehensive evaluations on continuity of care and optimising efficiency in management of chronic disease.

Few recent studies have considered management aspect of continuity of care in term of regularity of visiting GPs which captures the degree of regular contact with PHC providers [19, 20, 26]. Studies reported that regularity of contact is more important than the frequency of contact for reducing number and costs of hospitalisations [55, 56]. Greater regularity of visits more likely indicates care which is planned and proactive, while visits on an irregular basis (even if frequent/numerous) likely indicate care which is unplanned or reactive and thus not indicative of good ongoing management [43]. Current evidence also shows that use of the Enhanced Primary Care Medicare items increases regular PHC contact in the following year [26, 55], suggesting that regularity is suitable as a target for health policy intervention [25, 26].

Our new time-duration concept extends on the concept of regularity by adding a time component. This is important as care can be regular if a patient sees their GP once per year, but this might not be sufficient (i.e. the time-duration may be too long between visits) to provide adequate management of the patient’s condition and therefore some of the protective effects of regular care may be lost. Although the concept of time duration between services is relative new in health care services research, it has been integrated in other research areas such as customer relationship management [35, 40, 41, 46] and pharmaceutical studies to capture medication persistence – a proportion of time duration under adequate medication supply [9, 48]. Our new metric – the Cover Index is defined as the proportion of days, within a fixed ascertainment period (preferably 1 year since this is the time period that current chronic disease management plans are based [50]) that a patient is considered under the ‘protective effect’ of their PHC contact and at reduced risk of PPH. In contrast to drug utilisation studies where medication protective effect is well defined, in primary care, no data exist providing the duration over which a GP visit has the potential to protect a patient from an adverse event or complication of their chronic disease.

We hypothesise that interaction with a GP can protect a patient from experiencing a diabetes-related potentially preventable hospitalisation and that this protective effect can be maintained if GP interactions fall within a particular maximum time interval (i.e. do not exceed this time) named the “optimal maximum time interval”. Our study aimed to develop a methodology for determining “cover” of primary care using individual-level linked administrative data by (i) estimating the optimal maximum time interval over which primary care affords an increased protection from PPH using threshold effects models; and (ii) using the derived optimal time period to operationalise “cover” at the individual level.

## Methods

### Time-duration index of continuity of primary care (cover) development

**Fig. 1:**
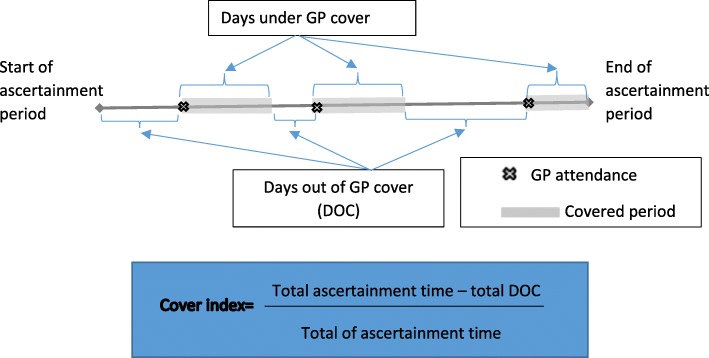
Calculation of cover index

The proposed time-duration index, which we call “Cover”, is defined as the proportion of time that an individual is under the potentially protective effect of PHC (via contact with their GP) over a pre-specified ascertainment period. Construction of the index relies on first determining a period of time between GP visits that a patient with a stated set of socio-demographic and clinical characteristics has a reduced probability of PPH. We term this the ‘optimal maximum time interval’. Once this optimal time period has been determined cover can be calculated as shown in Fig. 1. Briefly, the actual time interval (in days) between each GP attendance within the ascertainment period is first determined. This time is then compartmentalised into within and outside of the pre-defined optimal maximum time interval for persons with pre-defined characteristics in that year. The number of days within the optimal maximum time interval (i.e. days covered) are then aggregated over an ascertainment period for each individual in the complication cohort and the proportion of the total number of days eligible for cover over the ascertainment period calculated. This provides the cover index, which has a value between 0 and 1, for each individual in each year in our scenario (or some other time period chosen based on specific clinically or policy based rationale). A higher score reflects a greater proportion of time ‘covered’. Although methods used to calculate the cover score were demonstrated in complication cohorts of people with diabetes, the methods are applicable to other ambulatory care sensitive conditions.

### Estimation of the optimal time interval for GP services in people living with diabetes

#### Data sources

Western Australia (WA) whole-of-population administrative health data linked at the individual level for adults aged 18 years or older enrolled to vote in WA at any time between 1 July 1990 and 30 June 2004 were used for this study. The data included four datasets: WA Hospital Morbidity Data System (HMDS); Medicare Benefits Scheme (MBS) claim records; WA Electoral Roll (ER) records and WA mortality records. The HMDS provided information on diagnosis, date of admission and date of discharge from all WA hospitals. The MBS provided information on services provided outside the hospital (for example GP services) and included the date of service and type of medical service. The ER provided information on dates of migration in and out of WA or changes in a residential address while living in WA. Mortality records provided date and cause of death. WA data were linked and extracted via the WA Data Linkage System (WADLS)[33] and MBS data by the Commonwealth Department of Health and Ageing using a linkage key provided by the WADLS.

#### Study population

The study population consisted of people living with diabetes aged 45 years and older in WA for the years 1998/99 to 2003/04. Individuals with diabetes mellitus were determined using the International Classification of Disease, 9th edition-clinical modification (ICD-9-CM) codes in HMDS records and MBS claims indicative of the presence of diabetes using all the available data and has been described previously [31]. Three diabetes complication cohorts were constructed for this study depending on level of disease at each observed year: no diabetes complications, 1–2 complications and 3+ complications. Complication severity level was assessed using the complication severity index suggested by Young et al. [57] and stratified into three groups as outlined previously [31].

All individuals were observed annually from the baseline year to 30 June 2004, or last year living in WA or death with the data constructed as a panel (with years nested within a person). Only individuals who were alive and resident in WA for at least two consecutive years were included in the study. Individuals could move to a higher complication cohort if their complication status changed as ascertained at the end of each observed year. Within each complication cohort, we measured individual characteristics including GP utilisation, hospitalisations, complications, comorbidities and socio-demographic characteristics in each observed year, and GP utilisation and hospitalisations in the following year. A similar design has been applied in other studies [13, 31].

Ethical approval was provided by The University of Western Australia and Curtin University Human Research Ethics Committees.

#### Dependent variable

The number of diabetes-related potentially preventable hospitalisations during each follow-up year was the main outcome of the study. Diabetes-related hospitalisations were identified using ICD-9-CM and ICD-10-AM codes suggested by the National Health Performance Framework [1] and hospitalisations where diabetes was identified as a significant risk factor by Davis et al. [14].

#### Independent variables

GP utilisation including frequency of GP services and the time interval between GP services were focal measures in this study. For each individual, the date of GP services within a financial year was identified in MBS data. The time between GP visits was determined by number of days: (1) between GP visits within a financial year and; (2) between the date of first GP visit of a financial year and the date of the last GP visit in the previous financial year(s) looking back up to 3 financial years. In the case where a hospitalisation was observed, time was counted either to the first GP visit post-hospitalisation provided that the GP visit was within 14 days of discharge or from day 14 after hospital discharge date and the next GP visit [37]. The 14 day rule was applied based on a large scale study which suggests that timely follow-up within 14 days of discharge may be considered to reduce the risk of readmission for patient with multiple complex chronic conditions such as diabetes, heart disease and chronic obstructive pulmonary disease [37] and that time in excess of that would be deemed “out of cover”. The time intervals within a financial year were used to calculate the mean time interval for a GP visit, the variance of the time intervals and maximum time interval to a GP visit in months (or part thereof) of the financial year for each individual.

As mean time interval reflects central tendency of time intervals between services, two individuals can have the same mean time interval but their maximum time interval may be entirely different. In addition, the maximum time interval is more likely to capture the period of time that people were not covered by any protective effect of GP service contact than mean time interval. Thus, the maximum time interval to a GP visit in the following year was used as the main predictor of the number of hospitalisation in all analyses while mean time interval, frequency and regularity in the same year as well as mean time interval and regularity in the last year comprised covariates.

The variance of the time intervals was used to calculate the annual regularity of GP visits as [1/(1 + variance)] for each individual, described in detail elsewhere [19, 20, 26]. This regularity score was then converted into quintiles for each complication cohort. The frequency of GP usage was defined as a total number of GP visits within a financial year excluding those GP visits occurring within 14 days of the previous GP visit. This exclusion was to minimise over counting GP service utilisation as the visits within 14 days were thought by our expert primary care clinicians more likely to be associated with the existing episode of care rather than being indicative of a new episode (e.g. returning for the results of tests), recommended in the literature [17].

A number of individual socio-demographic and clinical characteristics were also measured. Demographic characteristics included were age groups (45–59 years, 60–74 years and ≥ 75 years), gender, and Indigenous status. Socio-economic status was assessed annually using quintiles of the Census specific Socio-Economic Indexes for Areas (SEIFA) Index of Relative Socioeconomic Disadvantage [4]. Service accessibility was measured annually classified as very remote, remote, moderate, accessible and highly accessible [3]. The number of comorbidities was summed using MACSS index [34], excluding conditions classified as complications of diabetes. Duration of diabetes was calculated in years from the first identification in WA linked data. Other use of health services was accounted for by capturing the number of specialist visits and the number of non-diabetes related hospitalisations in each financial year.

Average cost per hospitalisation was calculated and used to describe the characteristics of each complication cohort but not used as a controlling variable in regression models. Costs were assigned using Australian Refined Diagnostic Related Group costs from the National Hospital Cost Data collection and National Efficient Price of the Independent Hospital Pricing Authority [16]. All costs were adjusted to 2014 Australian dollar using the Consumer Price Index.

#### Statistical analyses

The data for each complication cohort were constructed as a panel data structure with multiple measures for each individual, such that response and control variables could vary over the study period. Panel data were complex and unbalanced as individuals could move in and out of WA, die or move to higher complication level cohort during the study period. Characteristics of the population were described for each complication cohort at the time entering to the cohort.

To estimate the optimal maximum time interval we employed threshold effects model proposed by (Gannon, Harris, and Harris 2014) to examine how the relationship between GP service and diabetes-related potentially preventable hospitalisation varies with the length of the time interval (the maximum time interval) between GP services. The model proceeded by searching for sample heterogeneity in the response of diabetes-related potentially preventable hospitalisation to variation in the time interval between GP services across populations in each complication cohort. The information criteria approach including Bayes Information Criterion (BIC) and Akaike Information Criterion (AIC) statistics was used to select the optimal model. The selected model was used to identify a number of subpopulations defined in terms of length of the time interval between GP services. The optimal model was used to suggest the maximum optimal time interval between GP services where the number of diabetes-related potentially preventable hospitalisations was minimal. The threshold effects model evaluates all subpopulations simultaneously rather than sequentially and therefore extends towards a non-linear model [22, 23, 27] that allows more flexibility in examining the relationship between GP service and the risk of diabetes-related potentially preventable hospitalisation. This approach has been applied in previous studies [24, 31].

The threshold effects model in our study was an extension of the random effects negative binomial model for panel data which accounts for time-variant factors and unbalance in the data structure. The general form of the model for individual *i* in year *t* presented as follows:

$$ {HOSP}_{it}=\sum \limits_{m=1}^M{\gamma}_m{R}_{m,i}\ast \Big({TInt}_{i,t}\ast {GPsvc}_{i,t}\left|{GPsvc}_{i,t}=1\&{TInt}_{i,t}\le 18\right)\kern0.5em +{\beta}_1\left(D1=1\ |{GPsvc}_{i,t}=0\right)+{\beta}_2\left(D2=1|\ {TInt}_{i,t}>18\right)\ast {Tint}_{i,t}+{\beta}_1{X}_{i,t}+{\beta}_2{\overline{x}}_{i,t}+{\alpha}_i+{HOSP}_{t0}+{U}_{i,t}+{\beta}_0 $$

$$ , i=1,2\dots N;t=1,2\dots, T $$

The equation is the hypothesised differential effect of GP services (*GPsvc*_*i*, *t*_) on diabetes-related PPH (*HOSP*_*it*_) with respect to an individual’s position with regard to the maximum time interval (*TInt*_*i*, *t* )_ to the next GP service. The threshold model allows the coefficient *γ*_*m*_ on GP service to vary according to the time interval to a GP service (in month) indicated by subpopulation indicators: *R*_*m*, *i*_ = 1 *if* {*τ*_*m* − 1_ < *Tint*_*i*, *t*_ ≤ *τ*_*m*_},   and 0 otherwise, where *m* is the number of subpopulation and *τ* is the threshold parameters. The number of subpopulation m (1, 2, 3 … M) and the threshold parameters *τ* was estimated from the data. The model splits the data into M subpopulations. The M = 1 setting gives the constant coefficient as a standard negative binomial model.

The threshold variable *R*_*i*_ only took values from 1 to 18 for two reasons: 1) 99% of the population in each complication cohort had a maximum time interval to a GP service ≤18 months, the sample size for the time interval > 18 months was relatively small (about 90 or less records for each time interval); 2) it was more computationally feasible as we could reduce the searching time. However, we still included the cases with the time interval > 18 months as a controlling variable (D2) with value of 1 if the maximum time interval > 18 months, and 0 otherwise.

The threshold effects model included a dummy variable (D1) for any observation with no GP service in a financial year to control for, rather than excluding, the observation. The model also included demographic and clinical characteristics in the observed years and GP utilisation in both observed and follow-up years in the notation *X*_*i*, *t*_ to control for any confounding. Endogeneity due to a correlation between the error term and the maximum time interval has been minimized by adding Mundlak variables *x̅*_*i*,*t*_, which are group means of time-varied variables including frequency of GP visits, regularity of GP visits and comorbidities. The group mean of time-varied variables relax the assumption of the random-effects estimator that unobserved factors were independent with the observed factors [11, 44]. In addition, the model also included initial conditions (history of hospitalisation at the baseline year, and GP utilisations in the previous years) to adjust for effects of unobserved heterogeneity [54].

All competing models were compared using their BIC and AIC statistics. The preferred model was the one which minimised the appropriate information criteria (AIC and BIC) [23]. Within each diabetes complication cohort, the preferred model indicated the maximum time interval to a GP service which had minimal risk of diabetes-related potentially preventable hospitalisations and suggested the maximum optimal time interval to a GP service corresponding to each diabetes complication cohort that was subsequently used to operationalise the cover index.

All analyses were conducted using STATA for Window version SE14.1.

### Operationalizing the cover index in the diabetes cohort

In this demonstration, the cover index was calculated for each financial year (July 1st to June 30th) for the studied period of 1998 to 2004, date of death or date of leaving WA which ever came first. The year of death was excluded from the analysis. Thus, part-years were not considered in the Cover Index calculation. For each financial year, the ascertainment days were the total number of days that people were living in the community (i.e. not in hospital).

Days out of GP cover (DOC) were calculated by subtraction of the pre-defined optimal maximum time interval (updated according to diabetes severity level) from the actual time interval between a GP service and the next health care service (either GP or hospital admission). Thus, by definition DOC values were positive. Any time interval that was shorter than the optimal maximum time interval was deemed as “under cover”, thus, DOC was counted as zero.

The cover index = [∑ascertainment days - ∑DOC] / ∑ascertainment days] was calculated for each individual annually. As the optimal maximum time interval was identified as a range of values from the threshold effects model, the cover index was calculated with low, middle and upper values bounds corresponding to low, middle and upper values of the optimal maximum time interval identified for each complication cohort (Fig. 1).

Values of cover were reported by socio-demographic characteristics of the cohort to explore the range of scores and serve to evaluate the face validity of the cover index in capturing vulnerable groups which traditionally have poor continuity of primary care.

## Results

### Characteristics of diabetes complication cohorts at the time entering the cohort

A total of 36,667 individuals aged 45 years or older were classified as living with diabetes in WA in this study. Since individuals could change complication cohorts (i.e. move to a higher complication group) throughout the study the total number of individuals shown in Table 1 reflects the number of individuals who were classified in that particular complication cohort at any time and is thus larger than the total number of individuals in the study. The complication cohorts are not mutually exclusive over the entire study period but are mutually exclusive within individual years (i.e. an individual cannot be in more than one complication cohort in the same financial year). During the studied period, 8968 individuals changed complication cohorts.

**Table 1:** Characteristics of studied population at the time entering each complication cohort

Characteristics	No complication	One or two complications	Three complications or more
	(N, (%))	(N, (%))	(N, (%))
N	20,039	14,866	10,730
Age group (years)			
45–59	9223 (46.0)	3849 (25.9)	1869 (17.4)
60–74	8650 (43.2)	7178 (48.3)	4708 (43.9)
≥ 75	2166 (10.8)	3839 (25.8)	4153 (38.7)
Gender			
Female	9741 (48.6)	7263 (48.8)	5000 (46.6)
Male	10,298 (51.4)	7603 (51.1)	5730 (53.4)
Indigenous status			
No	17,911 (95.9)	13,937(93.7)	9880 (92.1)
Yes	771 (4.1)	929 (6.2)	850 (7.9)
SEIFA			
Highest Disadvantage	3951 (19.8)	3232 (21.9)	2445 (22.9)
High disadvantaged	5540 (27.8)	4302(29.1)	3128 (29.3)
Moderate disadvantage	2792 (14.0)	2126 (14.4	1496 (14.0)
Less disadvantage	3205 (16.1)	2226 (15.1)	1582 (14.8)
Least disadvantage	4412 (22.2)	2876 (19.5)	2005 (18.8)
Accessibility			
Very remote	553 (2.8)	606 (4.1)	525 (4.9)
Remote	359 (1.8)	277 (2.0)	186 (1.7)
Moderate	945 (4.7)	772 (5.2)	603 (5.6)
Accessible	1039 (5.2)	843 (5.7)	627 (5.9)
Highly accessible	17,004 (85.4)	12,265 (83.0)	8716 (81.9)
Number of comorbidity			
Mean (SD)	3.0 (2.9)	5.7 (3.1)	8.3 (3.1)
Duration of diabetes (years)			
Mean (SD);	5.4 (3.8)	7.2 (4.2)	9.2 (4.7)
Regularity quantiles			
No regularity	4765 (23.8)	3150 (21.2)	2382 (22.2)
Quantile 1	3833 (19.1)	2776 (18.7)	2082 (19.4)
Quantile 2	3850 (19.2)	2873(19.3)	2070 (19.3)
Quantile 3	3772 (18.8)	2975 (20.0)	2116 (19.7)
Quantile 4	3819 (19.0)	3092 (20.8)	2080 (19.4)
Average time to a GP visit (months) Mean (SD)	3.6 (3.2)	2.8 (2.5)	2.4 (2.1)
Frequency of GP visits Mean (SD)	4.7 (2.8)	5.1 (3.0)	5.2 (3.2)
Number of specialist visits Mean (SD)	2.4 (4.1)	4.5 (6.5)	5.5 (9.2)
Number of non-diabetes related hospitalization	0.35 (1.57)	0.72 (2.3)	1.04 (1.85)
Number of diabetes related hospitalization	0.03 (1.06)	0.53 (1.7)	1.8 (10.8)
Average costs per diabetes related hospitalizations (2014 A$) (Mean (SD))	4381.2 (3828.7)	5185.5 (5492.7)	8192.7 (8992.6)
Min-Max	800.8–38,842.4	748.8–128,552.6	598.2–144,061.3
Average costs per non-diabetes related hospitalizations (2014 A$) (Mean (SD))	3993.3 (4132.2)	5637.4 (7964.4)	7756.2 (12,172.2)
Min-Max	393.4–61,680.1	393.4–142,694.4	662.2–227,080.1

Characteristics of the individuals at the time of entry into each complication cohort is presented in Table 1. Compare with individuals in the cohort with no complication, individuals in cohorts with higher complications were older (38.7% of those in three complication cohort and 25.8% of those in one or two complication cohort aged 75 years or older vs. 10.8% among those with no complication); had a higher number of comorbidities (average of 8.3 and 5.7 vs. 3.0 comorbidities, respectively); a longer duration of diabetes (9.2 years and 7.2 years vs. 5.4 years, respectively), a higher number of hospitalisations (1.8 and 0.53 hospitalisation per year vs. 0.03 hospitalisation per year, respectively) and higher average cost per hospitalisation (AU$ 7756.2 and AU$ 5637.4 per hospitalisation vs. AU$ 3993.2). However, other characteristics such as gender, socio-economic status and accessibility to services and GP usage did not vary between complication cohorts.

### Estimation of the optimal maximum time intervals for each diabetes complication cohort

**Fig. 2:**
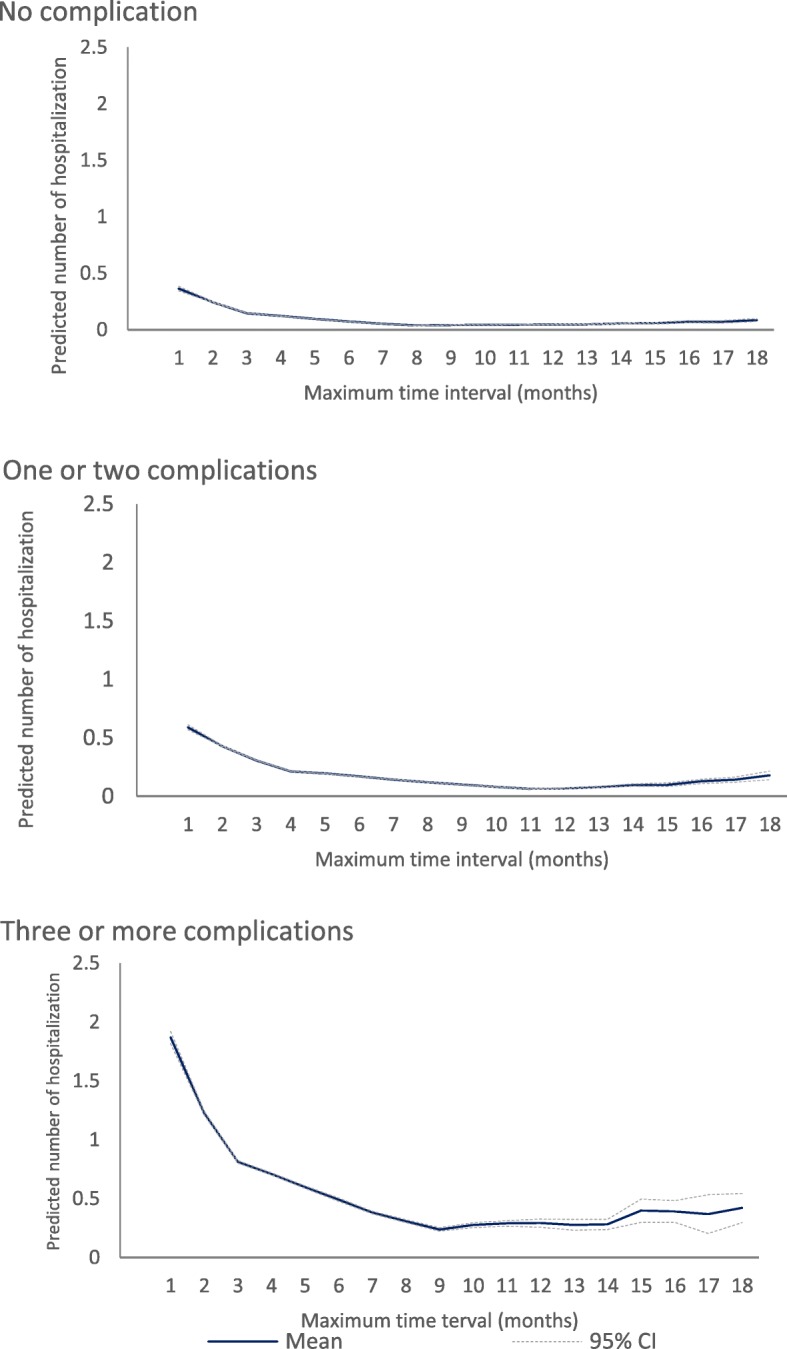
Changes in number of hospitalisations across maximum time interval between GP visits by complication cohort

Table 2 shows the results of the threshold effects model which presents how the relationship between GP service and the risk of diabetes-related PPH varies across the length of the maximum time interval between GP services by complication cohort. Based on both BIC and AIC, the preferred models indicated a non-linear relationship between maximum time interval between GP visits and the number of hospitalisations with five subpopulations in both no complication cohort and one or two complication cohort and four subpopulations in three or more complication cohort (Table 2). Overall, the expected number of diabetes related PPH was observed lowest in a maximum time interval between GP visits of 9 months to 13 months for diabetes with no complication; 5 months to 11 months for diabetes with one or two complications; and 4 months to 9 months for diabetes with three or more complications. For no complication cohort, the average number of predicted diabetes related potentially preventable hospitalisation within the optimal maximum time interval was 0.044 (95%CI, 0.043–0.045) admissions while the number was significantly higher among the sub-optimal time intervals (0.127 (95%CI, 0.126–0.128)). For one or two complication cohort, the average number of predicted diabetes related potentially preventable hospitalisation within the optimal maximum time interval was 0.159 (95%CI, 0.158–0.160) admissions while the number of hospitalisation was significantly higher among sub-optimal time interval (0.314 (95%CI, 0.311–0.316)). For three or more complication cohort, the predicted number of diabetes related potential preventable hospitalisations within the optimal maximum time interval was 0.589 (95%CI, 0.583–0.595) admissions while the number of hospitalisations was significantly higher among the sub-optimal time interval (1.15, 95%CI 1.14–1.16). The change in the number of predicted diabetes-related potentially preventable hospitalisations across the maximum time interval between GP visits using spline function is also presented in Fig. 2.

**Table 2:** Threshold search for max time to a GP visits by complications for people aged 45 years or older

Complication cohorts	No complication ^(a)^						One or two complications ^(b)^						Three or more complications©				
Number of subpopulations	2	3	4	5	6	18	2	3	4	5	6	18	2	3	4	5	18
AIC	45,169.0	45,091.2	45,049.5	45,020.4	45,017.6	45,018.7	53,464.2	53,413.6	53,360.2	53,346.5	53,408.3	53,340.3	69,875.6	69,812.3	69,774.9	69,769.4	69,777.3
BIC	45,398.5	45,329.9	45,297.3	45,277.4	45,283.8	45,450.1	53,683.6	53,641.8	53,597.2	53,592.3	53,662.8	53,752.9	70,086.1	70,031.2	70,002.3	70,005.2	70,173.0
Threshold parameters																	
τ_1_	8	8	2	2	2	–	10	2	2	2	3	–	9	2	2	2	–
τ_2_		13	8	3	3	–		10	3	3	6	–		3	3	3	–
τ_3_			13	8	8	–			11	4	7	–			9	9	–
τ_4_				13	10	–				11	9	–				13	–
τ_5_					13	–					11	–					
Coefficients																	
γ_1_	−0.229***	−0.249***	−0.040	0.134**	0.123**	–	−0.194***	−0.015	0.194***	0.323***	−0.165***	–	−0.128***	0.289***	0.196***	0.190***	–
	(0.01)	(0.01)	(0.03)	(0.05)	(0.05)	–	(0.01)	(0.03)	(0.04)	(0.05)	(0.03)	–	(0.01)	(0.03)	(0.03)	(0.03)	–
γ_2_	−0.083***	− 0.126***	− 0.199***	− 0.057	− 0.064*	–	− 0.053***	− 0.157***	0.006	0.087**	−0.216***	–	− 0.014	0.115***	0.054*	0.050*	–
	(0.01)	(0.01)	(0.02)	(0.03)	(0.03)	–	(0.01)	(0.01)	(0.02)	(0.03)	(0.02)	–	(0.01)	(0.02)	(0.02)	(0.02)	–
γ_3_		−0.013	−0.101***	−0.148***	− 0.152***	–		−0.039**	− 0.099***	−0.023	− 0.188***	–		−0.001	− 0.046**	−0.048**	–
		(0.01)	(0.01)	(0.02)	(0.02)	–		(0.01)	(0.01)	(0.02)	(0.02)	–		(0.01)	(0.02)	(0.02)	–
γ_4_			0.004	−0.072***	−0.089***	–			−0.000	− 0.071***	−0.162***	–			0.028*	0.013	–
			(0.01)	(0.01)	(0.02)	–			(0.01)	(0.02)	(0.02)	–			(0.01)	(0.01)	–
γ_5_				0.024	−0.057***	–				0.016	−0.116***	–				0.062***	–
				(0.01)	(0.02)	–				(0.01)	(0.02)	–				(0.02)	–
γ_6_					0.022	–					−0.039**	–					
					(0.01)	–					(0.01)	–					

For one subpopulation (a): AIC = 45,401.0; BIC = 45,676.3; (b) AIC = 53,622.8; BIC = 53,886.1; (c) AIC = 69,969.5; BIC = 70,222.1

### Cover index and its distributions

Table 3 shows the annual average cover index score overall for the whole studied population and by socio-demographic characteristics. Overall, the average cover score was 0.85 (upper bound) (95%CI 0.80 to 0.85) indicating that on average, in this cohort, 85% of the year people with diabetes were under the potentially protective effect of PHC via contact with their GP. However, only 83% of the time period was covered if the lower boundary of the optimal maximum time interval was considered rising to 84% of the time interval covered if the middle bound of the optimal maximum time interval was considered. The cover index score changed by socio-demographic characteristics. The lowest average cover index scores across low, middle and upper bounds was observed among those aged 75 years or older (0.77–0.78 - 0.79, respectively), males (0.80–0.82 - 0.83, respectively), indigenous (0.60–0.63 - 0.64, respectively), having highest disadvantage (0.81–0.82 - 0.83, respectively) and living in very remote areas (0.48–0.51 - 0.52, respectively).

**Table 3:** Average yearly cover score across maximal optimal time interval boundary over the studied period

Characteristics	Low bound cover		Middle bound cover		Upper bound cover	
	mean	95% CI	mean	95% CI	mean	95% CI
Overall						
Mean	0.83 (0.83; 0.83)		0.84	(0.84; 0.85)	0.85	(0.80; 0.85)
Median (IQR)	0.98 (0.81; 1.00)		1.00	(0.86; 1.00)	1.00	(0.87; 1.00)
Age group (years)						
45–59	0.82 (0.81; 0.82)		0.84	(0.83; 0.84)	0.85	(0.84; 0.85)
60–74	0.87 (0.87; 0.87)		0.88	(0.88; 0.89)	0.89	(0.89; 0.89)
≥ 75	0.77 (0.77; 0.77)		0.78	(0.78; 0.79)	0.79	(0.78; 0.79)
Gender						
Female	0.86 (0.86; 0.86)		0.87	(0.87; 0.87)	0.88	(0.88; 0.88)
Male	0.80 (0.80; 0.80)		0.82	(0.82; 0.82)	0.83	(0.83; 0.83)
Indigenous status						
No	0.84 (0.84; 0.84)		0.86	(0.86; 0.86)	0.86	(0.86; 0.86)
Yes	0.60 (0.59; 0.61)		0.63	(0.62; 0.64)	0.64	(0.63; 0.65)
SEIFA						
Highest Disadvantage	0.81 (0.80; 0.81)		0.82	(0.82; 0.83)	0.83	(0.83; 0.83)
High disadvantaged	0.83 (0.83;0.84)		0.85	(0.85; 0.85)	0.86	(0.85; 0.86)
Moderate disadvantage	0.83 (0.83; 0.84)		0.85	(0.85; 0.85)	0.86	(0.85; 0.86)
Less disadvantage	0.84 (0.84; 0.84)		0.86	(0.85; 0.86)	0.86	(0.86; 0.87)
Least disadvantage	0.84 (0.84; 0.84)		0.86	(0.85; 0.86)	0.86	(0.86; 0.87)
Accessibility						
Very remote	0.48 (0.47; 0.49)		0.51	(0.50; 0.52)	0.52	(0.51; 0.53)
Remote	0.74 (0.73; 0.76)		0.77	(0.76; 0.78)	0.78	(0.77; 0.79)
Moderate	0.79 (0.79; 0.80)		0.82	(0.81; 0.82)	0.82	(0.82; 0.83)
Accessible	0.81 (0.81; 0.82)		0.83	(0.83; 0.84)	0.84	(0.84; 0.85)
Highly accessible	0.85 (0.85; 0.85)		0.86	(0.86; 0.87)	0.87	(0.87; 0.87)

## Discussion

Our study aimed to develop and operationalise the cover index, a novel measurement of continuity of primary care that represents an improvement in existing measurements of regularity of primary care through accounting for a time-limited protective effect achieved from interaction with a GP. This study presented an empirical approach to estimate the optimal time period for GP cover in a diabetes patient population in order to demonstrate its operationalisation, however, we suggest that the cover index could be flexibly operationalised with a range of a priori optimal time periods, such as those based on expert opinion or clinical guidelines, if applicable, to aid in both the development and evaluation of policies incentivizing provider-patient interactions. Differences in the cover index score operationalised in this way could be used as to evaluate the impact of such opinion, guidelines or policy on potentially preventable hospitalisations. The tremendous growth in the availability and range of whole-of-population administrative health datasets provide opportunities to measure the performance of health systems and evaluate the impact of health policy. However, currently available metrics are limited in their sophistication regarding the domains within utilisation they capture. The cover metric would significantly contribute to the advancement of available methods for the analysis of these data.

In these data, the threshold model indicated the optimal maximum time interval of 9–13 months for diabetes without complication, 5–11 months for one or two complications and 4–9 months for three or more complication where the risk of hospitalisation was minimised. This finding is in line with the recommendation in primary care guidelines for diabetes [2, 51] which suggest people with diabetes should receive primary care at regular intervals of 3–12 months depending on the complexity of individual needs. In addition, our findings are consistent with growing evidence that optimised primary care use may improve health outcomes and reduce resources used [30, 58]. However, current evidence does not clearly indicate specific time intervals for different disease severity levels, which may limit the ability to effectively measure primary care performance and utilisation. In addition to facilitating the operationalisation of cover our findings provide an important insight into primary care needs of people with diabetes corresponding to their severity level that may provide evidence for improvement of primary care performance.

Recent studies show various approaches such as counting a number of GP services in the short term or long term prior hospitalisation [53] or visualizing the density of GP services [21] to examine utilisation of GP services. In countries where GPs are the gatekeepers to access for most medical services, using these approaches may not capture underutilisation of GP services. Our study suggests using the maximum time interval between health care services in examining the relationship with the risk of hospitalisation since the maximum time interval drives attention towards the “long overdue period” likely to reflect discontinuity of GP care and lost opportunities for early treatment in the primary care setting.

Results of the variation in the average cover score show disparities in GP cover that are associated with socio-economic disadvantage, even though the results are only exploratory. The results are consistent with the literature showing poor access to primary care services among people from the low socio-economic background, Indigenous, and living in remote areas [8] and thus provide some face validity that the cover score performs in the way expected. The results also provide a quantification of the disparities in GP cover that is important information to target health care resources and provide a tool to accurately quantify the improvement in primary care resulting from interventions. Given the high burden of hospitalisation, improvement in GP cover would offer a cost-effective opportunity to reduce the costs of hospitalisation, especially among those with multiple complications. While not explored in this paper, in addition to capturing periods that are not covered, the metric could also be adapted to capture periods of “over cover” and thus be used to measure over as well as under servicing.

### Strengths and limitation of the study

The major strength of our study was using a threshold effects model, an advanced and flexible approach to comprehensively estimate the optimal time interval for a GP visit. A further strength of this study is the large population and comprehensive range of linked databases used in the empirical analysis that allowed us to measure and control any changes in both outcomes and exposures over the studied period.

The cover metric developed in this paper does not incorporate the number of GPs or GP practices visited. Since the purpose of the metric is to determine the influence of the time between visits adjusting for other dimensions of continuity (e.g. via the usual provider index, the frequency of visits and the number of practices visited) in models would be superior to incorporating these dimensions of continuity in the metric. Therefore, inclusive measure of time duration in the design of the Cover Index is a strength, since using the cover metric with separate adjustment for other dimensions of continuity allows the impact of the time duration component to be separated from other components. This is more valuable to practitioners and policy makers than a metric that reports a combined impact. Interaction terms could be used for evaluating various combinations of dimensions if required.

Our study has some limitations to consider when interpreting the results. The empirical analyses were conducted using data from 1990 to 2004, hence it cannot provide evidence regarding current utilisation of GP services. However, for the purposes of this paper, which sought to develop and operationalise the cover metric, the lack of contemporaneous data is unimportant. The cover metric could have been developed solely using synthetic data; however the use of these historical data is a strength because they allowed us to develop the metric using real world relationships between GP visits and other covariates and also afforded us the opportunity undertake face validity of the metric during the development stage. In addition, the use of these historical data could be considered a strength because this particular time period incorporates a period in Australia with little intervention aimed at increasing provision of primary care for people with chronic conditions. Thus, the data in this period could, with appropriate control of confounding factors, provide the baseline needed to identify the incremental impact of policies aimed at supporting continuity of primary care, via changes in the cover score and associated impact on PPHs.

Administrative data are not collected for research purposes, hence, they do not include some details about severity of disease. Our data also did not have information about whether individuals visited the same or different GPs which may have improved the threshold modelling of our estimation of the maximum optimal time period. As the same provider is a potential factor for a holistic approach to continuity of care, future work may wish to expend on the current metric with inclusion of such data. The empirical results were limited to those who were clinically diagnosed with diabetes and incur health care resource utilisation through hospitals or Medicare claims that may affect generalisation of the maximal optimal time period estimated. Although the covered time interval found in our study relates only to diabetes at particular severity levels, the cover metric has application to other ambulatory care sensitive chronic conditions.

## Conclusions

Our study adds to the current literature by developing and operationalizing a new approach to measuring continuity of primary care which incorporates time-duration protective effects of primary care. This study used novel threshold modelling to determine the impact of maximum duration between GP services on preventable hospitalisation and used the estimated value to operationalise cover. However, the operationalisation of cover is flexible and allows for use of a priori time intervals, which makes it ideal to evaluate clinical guidelines and policies that recommend specified durations between GP visits.

## Abbreviations

AIC: Akaike Information Criterion
BIC: Bayes Information Criterion
COC: Continuity of care
DOC: Days out of Cover
ER: Western Australian Electoral Roll
GP: General Practitioner
HMDS: Hospital Morbidity Data System
MBS: Medicare Benefits Scheme
PHC: Primary health care
PPH: Potentially Preventable Hospitalisation
WA: Western Australia
WADLS: Western Australian Data Linkage System
